# Llamado a la implementación de la Ley 2120 de 2021 contra la comida chatarra en los tiempos del COVID-19 en Colombia

**DOI:** 10.7705/biomedica.6303

**Published:** 2022-10-31

**Authors:** Lina María García, Adolfo Contreras

**Affiliations:** 1 Grupo Pacífico Siglo XXI y CEDETES, Escuela de Odontología, Universidad del Valle, Cali, Colombia Universidad del Valle Universidad del Valle Cali Colombia; 2 Grupo Medicina Periodontal, Escuela de Odontología, Universidad del Valle, Cali, Colombia Universidad del Valle Universidad del Valle Cali Colombia

**Keywords:** infecciones por coronavirus, pandemias, alimentos, desnutrición, enfermedades no transmisibles, salud pública, Coronavirus infections, pandemics, food, malnutrition, noncommunicable diseases, public health

## Abstract

Durante dos años y medio, tanto en Colombia como en el resto del mundo, hemos enfrentado la pandemia generada por el SARS-CoV-2, agudizando los múltiples problemas de salud pública que venían cursando a manera de sindemias. Tal es el caso de las enfermedades crónicas no transmisibles asociadas, entre otros factores, a los malos hábitos alimenticios, especialmente por el gran consumo de alimentos ultraprocesados y bebidas azucaradas. En julio del 2021, se aprobó la Ley 2120, por medio de la cual se adoptaron medidas para fomentar entornos alimentarios saludables.

Entre estas medidas, también se promueve que los alimentos empacados, procesados y que afectan negativamente la salud humana, tengan un sello que identifique sus componentes y valor nutricional para informar a los consumidores sobre el producto que están comprando o consumiendo.

Entre las enfermedades más prevalentes en Colombia que afectan la salud bucal, se encuentran las caries y las periodontitis, ambas con factores de riesgo comunes con las enfermedades crónicas no transmisibles. Dados sus costos y prevalencia, dichas enfermedades crónicas no transmisibles resultan primordiales desde un enfoque de gestión del riesgo en salud pública, siendo, quizá, su gravedad mayor durante la pandemia generada por el SARS CoV-2.

Asimismo, se especula que la población confinada pudo llegar a consumir más comida chatarra durante la pandemia del COVID-19 en comparación con otros períodos, además, porque en aquellos con enfermedades crónicas no transmisibles, el confinamiento obligado se asocia con mayor sedentarismo y con un menor número de controles médicos regulares, asuntos que se han reportado previamente.

La promulgación de la ley de comida chatarra no va a cambiar los hábitos de alimentación de los colombianos “de la noche a la mañana” y, por esto, se necesita con urgencia implementar procesos de educación y sensibilización frente a los efectos adversos de los alimentos procesados y ultraprocesados en la salud.

## Abstract (en)

For more than one year and a half, Colombia and the world have been facing the effects caused by the pandemics of SARS CoV-2 with concomitant and aggravating deterioration of multiple public health problems such as noncommunicable diseases. Noncommunicable diseases are frequently associated with poor eating habits and regular consumption of processed and ultra-processed foods, among other important risk factors related to culture, genetics, and sedentarism.

In July, 2021, Colombia ratified the Law 2120 that promotes healthy food habits and recreational environments, by forcing food producers to include nutritional advertisement seals on processed food packages, with explanatory guidelines for consumers about calories and nutritional value.

This editorial note highlights the importance in Odontology of promoting and implementing this Law through education and sensitization of populations, in order to cope with some of the adverse risk factors of the hypercaloric diet.

En la última semana de julio del 2021, en Colombia se aprobó la Ley 2120 contra la comida chatarra, que promueve que los alimentos empacados, procesados y que afectan la salud humana, tengan sellos que identifiquen sus componentes y que revelen su valor nutricional para ilustrar a los consumidores sobre el producto que están comprando o consumiendo ^1^. Estos alimentos procesados y ultraprocesados normalmente aportan muchas calorías por su gran contenido de azúcares, grasas saturadas y sodio, y su contenido en fibra y en micronutrientes es poco o nulo. Además, estos supuestos alimentos procesados y ultraprocesados incorporan conservantes y saborizantes, como las sales de nitro y nitritos, y reforzadores del sabor, que inducen un consumo frecuente y desmesurado, lo cual incrementa el riesgo de sufrir sobrepeso y obesidad, así como de enfermedades crónicas no transmisibles ^2^.

En Colombia, la industria de alimentos informa en los empaques la cantidad de grasa, azúcar, sodio y calorías que contienen los productos, pero este etiquetado es insuficiente, puesto que la mayoría de los compradores desconocen cuál porcentaje de estos componentes es dañino, y cuál es el valor nutricional según las porciones ^1^. Desde mediados de marzo del 2020, el país se ha enfrentado a la emergencia sanitaria debida al virus SARS CoV- 2, generándose un relativo confinamiento por ciudades y regiones, en el cual se especula que ha habido un incremento de hábitos de mala alimentación y de sedentarismo que preocupan a las autoridades de salud pública ^2^^-^^4^. En el estudio ENSIN-2020 realizado en el país, se reportó que el 56 % de los adultos en Colombia presenta sobrepeso y obesidad, y, además, que el sedentarismo ha venido aumentando entre los niños, los jóvenes y las mujeres ^5^. Esta situación puede haberse agravado con las medidas restrictivas interpuestas, y por el acceso restringido a los sitios de educación y trabajo, así como a gimnasios, piscinas, vías, parques y otros sitios para hacer deporte y recreación.

La comida chatarra más consumida en Colombia incluye: productos empaquetados -como papa, plátano y yuca fritos-, galletas, helados, chocolates y caramelos, productos de panadería y repostería, cereales, barras “energizantes”, mermeladas y jaleas, bebidas maltosas, gaseosas y azucaradas, y bebidas de chocolate; además, productos procesados y ultraprocesados listos para calentar o comer, tanto en el hogar como en locales de comida rápida, en donde es fácil encontrar preparaciones como pizzas, hamburguesas, mazorcadas y perros calientes, productos cárnicos apanados, pastas y postres en polvo o envasados, entre otros ^6^.

Uno de los problemas asociados con la comida chatarra es que se ingiere a cualquier hora del día, sin importar los horarios usuales de alimentación, al estar disponible para los consumidores las 24 horas de los 7 días de la semana. Asimismo, estos productos incorporan saborizantes que los hacen adictivos. Estos dañinos atributos de la comida chatarra predisponen a su consumo exagerado y promueve una dieta hipercalórica por alimentos procesados y ultraprocesados, por lo cual se asocian con la pandemia de las enfermedades crónicas no trasmisibles ^7^.

La situación puede agravarse aún más con las medidas de control sanitario que pretenden limitar la propagación del SARS CoV-2, que favorecen un mayor consumo de comida chatarra a causa del sedentarismo y el encierro, y mayores niveles de tensión emocional, que se asocian con una menor disponibilidad de espacios para disfrutar al aire libre.

Finalmente, los comerciantes de la comida chatarra utilizan la televisión, la radio, la prensa y los mensajes de texto por medio de teléfonos y otras aplicaciones digitales, para promover dicho consumo en todos los segmentos de la población, impactando, asimismo, en niños y jóvenes. Por el contrario, aquellos segmentos publicitarios asociados con las políticas del gobierno y del Ministerio de Salud que promueven una sana alimentación acompañada de actividad física, poseen canales de distribución y tiempos limitados en comparación con los que promueven alimentos procesados ^8^.

## Consumo en Latinoamérica y en Colombia

En Latinoamérica, el consumo de azúcar es el doble de lo recomendado por la Organización Mundial de la Salud (OMS), con una media de de 99,4 g al día, cuando la recomendación es de 52 g en este este mismo lapso ^9^^,^^10^. La ingestión media de bebidas azucaradas en adultos en el mundo fue de 226 ml al día, siendo Latinoamérica la región con mayor consumo. México es el líder del consumo de gaseosas azucaradas a nivel global y posee, también, la más elevada tasa mundial de prediabetes y diabetes, a pesar de tener un impuesto destinado a controlar el consumo de estas bebidas. Esto se explica por la complejidad de los cambios de hábitos asociados con los valores sociales y las costumbres alimenticias, y a la cultura de las diferentes poblaciones. Es así como, en contraste, en Suecia se consume más agua y leche ^10^.

Según la Organización Panamericana de la Salud (OPS), debido al alto consumo de productos ultraprocesados y dañinos para la salud en Colombia, una dieta poco saludable es el segundo factor de riesgo asociado con el incremento de la morbilidad y mortalidad ^4^. Nueve de cada diez escolares no cumplen con la frecuencia de consumo de frutas y verduras recomendado por la OMS. Asimismo, el 82,4 % de estos niños consume productos de paquete, por lo menos, en uno de los siete días de la semana, y uno de cada dos consume comidas rápidas ^11^^,^^12^.

## Enfermedades asociadas

Diversas enfermedades se han asociado con el excesivo consumo de alimentos y bebidas azucaradas tales como la diabetes, la obesidad mórbida, la enfermedad cardiovascular, el cáncer, el síndrome metabólico, la enfermedad renal crónica, asimismo, diversos problemas musculoesqueléticos y la caries ^13^.

En Colombia en los últimos 10 años, el sobrepeso y la obesidad se han incrementado en un 10 %, porcentaje similar al de consumo de bebidas gaseosas azucaradas ^10^. El exceso de peso en niños en edad escolar (5 a 12 años) se incrementó anualmente en un 1 % entre el 2010 y el 2015. El consumo de dos o más vasos diarios de bebidas ultraprocesadas, puede incrementar en 52 % el riesgo de morir a causa de infarto cardiaco, hipertensión arterial sistémica o derrame cerebral, entre otras condiciones.

Estos efectos acumulativos se hacen más evidentes entre la quinta y la sexta décadas de la vida ^5^^,^^14^. El consumo de productos procesados y ultraprocesados por niños en edad escolar se ha asociado con menor calidad nutricional y bajos niveles de ácidos grasos omega 3, vitaminas A, B12, C y E, así como de calcio y cinc ^15^. El consumo de este tipo de productos incrementa el riesgo de desarrollar diabetes, independientemente del peso corporal ^16^, así como el consumo de bebidas azucaradas incrementa hasta en un 28 % la incidencia de diabetes mellitus de tipo 2 ^17^.

Según el IV Estudio Nacional de Salud Bucodental (ENSAB-IV), el 91,58 % de las personas entre 12 y 79 años ha presentado caries dental en algún momento de su vida. De igual forma, el 33,27 % de los niños de 1, 3 y 5 años, ha tenido, a su corta edad, caries asociadas con el consumo excesivo de azúcares, y con fallas en la remoción frecuente y eficaz de la placa dental ^18^. Muchos padres de familia y cuidadores de niños en edad preescolar y escolar, no comprenden que la caries de la infancia temprana y en diferentes edades, se asocia con el consumo excesivo y habitual de bebidas azucaradas, como el agua de panela, y con rutinas de higiene bucal deficientes ^19^.

## Implicaciones de la ley contra la comida chatarra

La Ley 2120 del 2021 pretende reducir las enfermedades crónicas más prevalentes, como la diabetes, la caries dental, las cardiovasculares y las cerebrovasculares, disminuyendo el consumo de azúcares, grasas y sal, y mejorando las capacidades de “compra y consumo responsable” de alimentos orientadas a una mejor selección de productos. La Ley debe ir acompañada de un proceso educativo a partir de estrategias que contengan información y actividades que refuercen los buenos hábitos y los estilos de vida saludables. Asimismo, se deben fomentar el deporte y la recreación, así como proponer pautas y acciones relacionadas con el seguimiento de los indicadores de las enfermedades no transmisibles y la promoción de entornos saludables, especialmente en niños y adolescentes. Estas propuestas serán lideradas por la Comisión Intersectorial de Seguridad Alimentaria y Nutricional (CISAN) ^1^.

Además, la Comisión de Regulación de Comunicaciones debe autorizar espacios institucionales en los canales de televisión abierta para promover hábitos saludables en horarios “pico”, lo cual se requiere para complementar el efecto que se espera que produzca la Ley 2021. Por otro lado, el Instituto Nacional de Vigilancia de Medicamentos y Alimentos (INVIMA) debe asumir las actividades de inspección, vigilancia y control de lo dispuesto por esta Ley, así como controlar que la información sea verídica y que corresponda con el contenido real de los productos ^1^.

Se deben crear y promover entornos saludables en todos los espacios educativos y de recreación, que incluyan el consumo de agua potable, frutas y verduras, y una alimentación balanceada y saludable. De igual forma, se debe fomentar la actividad física e impulsar estrategias educativas para interpretar adecuadamente el etiquetado nutricional de los empaques.

Así como el Gobierno Nacional gestionó la promulgación de esta Ley es necesario avanzar en su reglamentación e implementación mediante un trabajo articulado con los profesionales de la salud y su inclusión en los planes de estudio. Igualmente, se requiere una articulación intersectorial entre los ministerios de Industria, Agricultura, Deporte y Recreación, Salud y Protección Social, y el de Educación Nacional, con el fin de poder garantizar una salud integral y mayor bienestar para la población colombiana.
